# A systematic review of musculoskeletal disorders among school teachers

**DOI:** 10.1186/1471-2474-12-260

**Published:** 2011-11-17

**Authors:** Patience N Erick, Derek R Smith

**Affiliations:** 1 School of Health Sciences, University of Newcastle, 10 Chittaway Road, Ourimbah, 2258, Australia

## Abstract

**Background:**

Musculoskeletal disorders (MSD) represent one of the most common and most expensive occupational health problems in both developed and developing countries. School teachers represent an occupational group among which there appears to be a high prevalence of MSD. Given that causes of MSD have been described as multi-factorial and prevalence rates vary between body sites and location of study, the objective of this systematic review was to investigate the prevalence and risk factors for MSD among teaching staff.

**Methods:**

The study involved an extensive search of MEDLINE and EMBASE databases in 2011. All studies which reported on the prevalence and/or risk factors for MSD in the teaching profession were initially selected for inclusion. Reference lists of articles identified in the original search were then examined for additional publications. Of the 80 articles initially located, a final group of 33 met the inclusion criteria and were examined in detail.

**Results:**

This review suggests that the prevalence of self-reported MSD among school teachers ranges between 39% and 95%. The most prevalent body sites appear to be the back, neck and upper limbs. Nursery school teachers appear to be more likely to report suffering from low back pain. Factors such as gender, age, length of employment and awkward posture have been associated with higher MSD prevalence rates.

**Conclusion:**

Overall, this study suggests that school teachers are at a high risk of MSD. Further research, preferably longitudinal, is required to more thoroughly investigate the issue of MSD among teachers, with a greater emphasis on the possible wider use of ergonomic principles. This would represent a major step forward in the prevention of MSD among teachers, especially if easy to implement control measures could be recommended.

## Background

Musculoskeletal disorders (MSD) represent one of the most common and important occupational health problems in working populations, being responsible for a substantial impact on quality of life and incurring a major economic burden in compensation costs and lost wages [1,2]. MSD decrease productivity at work due to sick leave, absenteeism and early retirement [3-5], and are also costly in terms of treatment and individual suffering [6]. Moreover, MSD represent a common health-related reason for discontinuing work and for seeking health care. In many occupations, MSD include a wide range of inflammatory and degenerative conditions affecting the muscles, ligaments, tendons, nerves, bones and joints; and can occur from a single or cumulative trauma [7,8].

The work tasks of school teachers often involves significant use of a 'head down' posture, such as frequent reading, marking of assignments, and writing on a blackboard [9,10]. Nursery school teachers, however, also perform a wide variety of tasks combining basic health childcare and teaching duties, and those that require sustained mechanical load and constant trunk flexion. Nursery school teachers have been found to have elevated prevalence of neck, shoulder, arm and low back disorders [11,12], and lower-extremity MSD due to activities which require sustained periods of kneeling, stooping, squatting or bending [11].

School teachers, in general, have been demonstrated relative to other occupational groups, to report a high prevalence of MSD [3], with prevalence rates of between 40% and 95% [3,7,13-17]. During the course of their work, teachers may be subjected to conditions that cause physical health problems [14]. The work of a teacher does not only involve teaching students, but also preparing lessons, assessing students' work and being involved in the extracurricular activities such as sports. Teachers also participate in different school committees. These may cause teachers to suffer adverse mental and physical health issues due to the variety of job functions [14]. Despite this, the impact of MSD specifically within the teaching profession has not been given sufficient attention in the literature. Furthermore, comparatively little research has investigated the prevalence of MSD in the teaching profession.

The aim of this review was therefore, to critically analyse the literature and report on the prevalence of MSD and possible associated risk factors in the teaching profession. The review focused on nursery, primary and secondary school teachers and teachers of physically and mentally handicapped children.

## Methods

### Criteria for inclusion and exclusion

Empirical research, case studies and literature reviews published in peer reviewed English journals were considered for inclusion, with letters to the Editor and conference proceedings excluded. Participants in the studies had to have been listed as school teachers. No restrictions were placed on age, gender, race or socioeconomic status. Only articles that documented the prevalence of MSD and its risk factors were considered. Articles not written in English were excluded from the literature review, as were studies which reported on university academic staff.

### Search methods

An extensive literature search was undertaken in MEDLINE and EMBASE databases during 2011. Further searches were performed in occupational health and safety databases such as CISILO database and MAK Collection for Occupational Health and Safety. Other relevant databases that were searched for publications included AMED, CINAHL, Scopus, ProQuest and PubMed. Following the initial database search, the reference lists of articles initially identified were then examined for additional publications. Keywords used for the search were; musculoskeletal disorders, musculoskeletal discomfort, back pain and teachers.

### Study selection

For all research articles identified during the search, the titles, keywords and abstracts, where available, were considered for possible relevance to this literature review. Full text copies were obtained for analysis and data extraction for all articles that met the inclusion criteria.

### Search results

Following a thorough search of the databases, a total of 80 articles were located, albeit with a number of titles having been duplicated. The titles, keywords and abstracts (where available) were examined for relevance and assisted in the exclusion of duplicates. Following this process, a total of 40 potentially relevant papers were obtained. After further analysis of these articles, seven papers were excluded from the review as they did not measure the prevalence of MSD or demonstrate possible MSD risk factors among teachers. Articles that did not describe a research study or literature review were also excluded from the review. Following exclusions, a final group of 33 articles were considered suitable for the review.

## Results

### Description of studies

**Figure 1:**
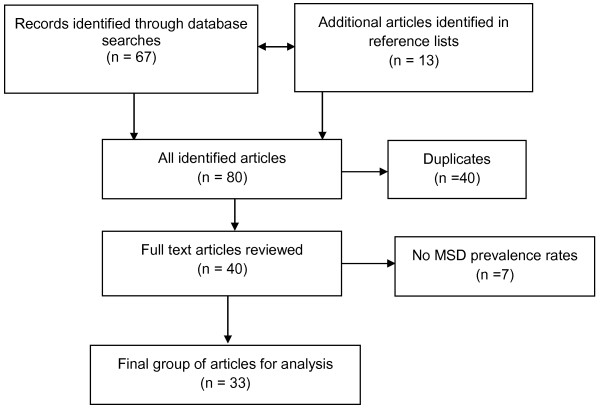
**Flow chart of the literature search**. The figure illustrates the details of the strategy used for literature search.

The 33 studies located during this review had either measured the prevalence of MSD or reported on possible risk factors for MSD among teachers. All studies had been published in English. Figure 1 provides a flow chart of the literature search methodology.

### Prevalence of MSD

International studies on MSD among school teachers have reported a high prevalence of MSD as indicated in Table 1. A number of articles reported a high prevalence of MSD, generally. A study of school teachers in Hong Kong, for example, found that 95.1% had experienced some form of pain in the previous month [14]. In a study carried out in Estonia which looked at physical activity, MSD and cardiovascular risk factors in male physical education teachers (PETs), 66.7% of teachers reported MSD in the previous 12 months, compared to 51.2% of PETs who reported MSD for the same period [16]. Furthermore, a study of Swedish music teachers found that 92% had experienced some form of pain in the previous 12 months [18], and a study of United States (US) music teachers found that 91% had experienced MSD [17]. In another Swedish study, 40% of school teachers and nursery school teachers were found to have reported MSD [13].

**Table 1:** International studies reporting the prevalence of MSD among school teachers

Body Site	Prevalence (%)	**Recall Period **^**a**^	Participants	Sample size	**Response Rate (%) **^**b**^	Country	**Year **^**c**^	**Study Design **^**d**^	Author (s)
Any	51.4	NS	Primary, secondary and high school teachers		900*	Turkey	2011	CS	Korkmaz *et al *[15]
	95.1	1 month	Primary and secondary school teachers	6 000	28.5	China	2010	CS	Chong & Chan [14]
	68.0	NS	Music teachers	580	87.1	Australia	2010	CS	Allsop & Ackland [7]
	55.0	NS	Primary and secondary school teachers	4 697	95.1	Brazil	2009	CS	Cardoso *et al *[3]
	77.0	12 months	Music teachers	61	77	Sweden	2009	CS	Edling & Fjellman-Wiklund [20]
	91.0	NS	Music teachers	1 600	3.5	US	2008	CS	Yoshimura *et al *[17]
	42.0	NS	School teachers	100	100	Germany	2005	CS	Seibt *et al*[33]
	82	12 months	Music teachers	287	72.5	Sweden	2003	CS	Fjellman-Wiklund *et al *[19]
	66.7	12 months	School teachers	359	74.6	Estonia	2002	CC	Pihl *et al*[16]
	51.2	12 months	Physical education teachers	359	74.6	Estonia	2002	CC	Pihl *et al*[16]
	40.0	NS	Nursery school teachers		224*	Sweden	1998	CS	Brulin *et al*[13]
	40.0	NS	School teachers		510*	Sweden	1998	CS	Brulin *et al*[13]
	92.0	12 months	Music teachers	61	58.1	Sweden	1998	CS	Fjellman-Wiklund & Sundelin [18]
	80.0	12 months	Music teachers	62	98.4	Sweden	1998	CS	Fjellman-Wiklund & Sundelin [18]
	78.0	NS	Preschool teachers	22	95.4	US	1995		Grant *et al*[11]
Neck only	42.5	NS	Primary, secondary and high school teachers		900*	Turkey	2011	CS	Korkmaz *et al *[15]
	68.9	1 month	Primary and secondary school teachers	6 000	28.5	China	2010	CS	Chong & Chan [14]
	47.0	12 months	Music teachers	61	77	Sweden	2009	CS	Edling and Fjellman-Wiklund [20]
	69.3	Life-long	Secondary school teachers	5 680	54.6	China	2007	CS	Chiu & Lam [9]
	66.7	12 months							
	59.7	Since becoming a teacher							
	68.2	Life-long	Secondary school teachers	1 500	44.8	China	2006	CS	Chiu *et al *[6]
	64.4	12 months							
	56.8	Since becoming a teacher							
	59.0	12 months	Music teachers	287	72.5	Sweden	2003	CS	Fjellman-Wiklund *et al *[19]
	30.0	7 days							
	33.3	12 months	School teachers	359	74.6	Estonia	2002	CS	Pihl *et al*[16]
	9.3	12 months	Physical education teachers	359	74.6	Estonia	2002	CS	Pihl *et al*[16]
	44.4	12 months	Music teachers	61	58.1	Sweden	1998	CS	Fjellman-Wiklund & Sundelin [18]
	38.9	12 months	Music teachers	62	98.4	Sweden	1998	CS	Fjellman-Wiklund & Sundelin [18]
Shoulder only	28.7	NS	Primary, secondary and high school teachers		900*	Turkey	2011	CS	Korkmaz *et al *[15]
	73.4	1 month	Primary and secondary school teachers	6 000	28.5	China	2010	CS	Chong & Chan [14]
	28.0	12 months	Music teachers	61	77	Sweden	2009	CS	Edling *et al*[20]
	55.0	12 months	Music teachers	287	72.5	Sweden	2003	CS	Fjellman-Wiklund *e al*[19]
	31.0	7 days							
	7.8	12 months	School teachers	359	74.6	Estonia	2002	CC	Pihl *et al*[16]
	18.6	12 months	Physical education teachers	359	74.6	Estonia	2002	CC	Pihl *et al*[16]
	55.6	12 months	Music teachers	61	58.1	Sweden	1998	CS	Fjellman-Wiklund & Sundelin [18]
	38.9	12 months	Music teachers	62	98.4	Sweden	1998	CS	Fjellman-Wiklund & Sundelin [18]
	22	After being on duty	Nursery school teachers	1 059	73	Japan	1981	CS	Nagira *et al *[32]
Neck and/or shoulder	25 - 35.4	1 month	Nursery school teachers	1 445	99.5	Japan	2002	CS	Ono *et at*[21]
	33.0		Preschool teachers	22	95.4	United States	1995		Grant *et al*[11]
Upper limbs	8.0 (elbows)	NS	Primary, secondary and high school teachers		900 respondents	Turkey	2011	CS	Korkmaz *et al *[15]
	13.0 (wrist only)	NS	Primary, secondary and high school teachers		900*	Turkey	2011	CS	Korkmaz *et al *[15]
	43.9 (arm)	I month	Primary and secondary school teachers	6 000	28.5	China	2010	CS	Chong & Chan [14]
	23.7	NS	Primary and secondary school teachers	4 697	95.1	Brazil	2009	CS	Cardoso *et al *[3]
	19.0 (elbows)	12 months	Music teachers	61	77	Sweden	2009	CS	Edling *et al*[20]
	15.0 (hands)	12 months	Music teachers	61	77	Sweden	2009	CS	Edling *et al*[20]
	35.8	Life-long	Secondary school teachers	5,680	54.6	China	2007	CS	Chiu & Lam [9]
	33.3	12 months							
	31.8	Since becoming a teacher							
	72.1	NS	Teachers for physically and intellectually disabled pupils	1 663	84.8	Japan	2003	CS	Yamamoto *et al*[22]
	18.0 (elbows)	12 months	Music teachers	287	72.5	Sweden	2003	CS	Fjellman-Wiklund *et al *[19]
	8.0 (elbows)	7 days							
	20.0 (hands)	12 months	Music teachers	287	72.5	Sweden	2003	CS	Fjellman-Wiklund *et al *[19]
	13.0 (hands)	7 days							
	9.1 - 17.7 (arms)	1 month	Nursery school teachers	1 445	99.5	Japan	2002	CS	Ono *et al*[21]
	22.2 (elbows)	12 months	Music teachers	61	58.1	Sweden	1998	CS	Fjellman-Wiklund & Sundelin [18]
	22.2 (hands)	12 months	Music teachers	61	58.1	Sweden	1998	CS	Fjellman-Wiklund & Sundelin [18]
	11.1 (elbows)	12 months	Music teachers	62	98.4	Sweden	1998	CS	Fjellman-Wiklund & Sundelin [18]
	19.4 (hands)	12 months	Music teachers	62	98.4	Sweden	1998	CS	Fjellman-Wiklund & Sundelin [18]
	11.0 (hand/wrist)	NS	Preschool teachers			US	1995		Grant *et al*[11]
Back	36.9 (upper back)	NS	Primary, secondary and high school teachers		900*	Turkey	2011	CS	Korkmaz *et al *[15]
	43.8 (lower back)	NS	Primary, secondary and high school teachers		900*	Turkey	2011	CS	Korkmaz *et al *[15]
	52.5 (upper back)	1 month	Primary and secondary school teachers	6 000	28.5	China	2010	CS	Chong & Chan [14]
	59.2 (lower back)	1 month	Primary and secondary school teachers	6 000	28.5	China	2010	CS	Chong & Chan [14]
	40.4	12 months	Primary school teachers		272*	Malaysia	2010	CS	Samad *et al*[1]
	32.0 (upper back)	12 months	Music teachers	61	77	Sweden	2009	CS	Edling *et al*[20]
	49.0 (lower back)	12 months	Music teachers	61	77	Sweden	2009	CS	Edling *et al*[20]
	41.1	NS	Primary and secondary school teachers	4 697	95.1	Brazil	2009	CS	Cardoso *et al *[3]
	52.4	NS	Teachers in a special school for the severe handicaps	50	88		2009	CS	Wong *et al*[24]
	53.3	NS	Secondary school teachers	992	52.2	Philippines	2007	CS	Atlas *et al*[8]
	34.8	6 months	School teachers	1 869	52.1	France	2006	CS	Kovess- Masfety *et al *[28]
	45.7	1 month	School teachers for physically and mentally handicapped children	1 869	56.3	Japan	2006	CS	Muto *et al*[25]
	63.0	NS	Physical education teachers		562*	Greece	2004	CS	Stergioulas *et al *[23]
	50.0	Life-long	Primary school teachers	492	78	China	2004	CS	Jin *et al*[27]
	40.0	12 months							
	22.0	1 week							
	35.0 (upper back)	12 months	Music teachers	287	72.5	Sweden	2003	CS	Fjellman-Wiklund *et al *[19]
	21.0 (upper back)	7 days							
	45.0 (lower back)	12 months	Music teachers	287	72.5	Sweden	2003	CS	Fjellman-Wiklund *et al *[19]
	23.0 (lower back)	7 days							
	76.7	NS	Teachers for physically and intellectually disabled pupils	1 663	84.8	Japan	2003	CS	Yamamoto *et al *[22]
	4.7 (lower back)	12 months	Physical education teachers	359	74.6	Estonia	2002	CC	Pihl *et al*[16]
	11.8 (lower back)	12 months	School teachers	359	74.6	Estonia	2002	CC	Pihl *et al*[16]
	43.0	12 months	Nursery school teacher	10 351	62.7	Japan	2002	CS	Tsuboi *et al*[5]
	20.6	12 months	Elementary, junior and senior high school teachers	10 351	62.7	Japan	2002	CS	Tsuboi *et al*[5]
	33.3 (upper back)	12 months	Music teachers	61	58.1	Sweden	1998	CS	Fjellman-Wiklund & Sundelin [18]
	55.6 (lower back)	12 months	Music teacher	61	58.1	Sweden	1998	CS	Fjellman-Wiklund & Sundelin [18]
	50.0 (lower back)	12 months	Music teacher	62	98.4	Sweden	1998	CS	Fjellman-Wiklund & Sundelin [18]
	25.0 (upper back)	12 months	Music teachers	62	98.4	Sweden	1998	CS	Fjellman-Wiklund & Sundelin [18]
	17.7	1 month	Nursery school teachers	2 829	98.9	Japan	1997	CS	Ono *et al*[26]
	61.0	NS	Preschool teachers	22	95.4	US	1995		Grant *et al*[11]
	39.2 (low back pain)	1 month	Nursery school teachers	1 059	73	Japan	1981	CS	Nagira *et al*[32]
Lower limb/extremities	8.4 (hip)	NS	Primary, secondary and high school teachers		900*	Turkey	2011	CS	Korkmaz *et al *[15]
	32.0 (knees)	NS	Primary, secondary and high school teachers		900*	Turkey	2011	CS	Korkmaz *et al *[15]
	21.8 (ankles)	NS	Primary, secondary and high school teachers		900*	Turkey	2011	CS	Korkmaz *et al *[15]
	54.6 (leg pain during physical activity)	1 month	Primary and secondary school teachers	6 000	28.5	China	2010	CS	Chong & Chan [14]
	41.1	NS	Preschool & primary school teachers	4 697	95.1	Brazil	2009	CS	Cardoso et al [3]
	12.0 (hips)	12 months	Music teachers	287	72.5	Sweden	2003	CS	Fjellman-Wiklund *et al *[19]
	4.0	7 days							
	16.0 (knees)	12 months	Music teachers	287	72.5	Sweden	2003	CS	Fjellman-Wiklund *et al *[19]
	5.0	7 days							
	9.0 (feet)	12 months	Music teachers	287	72.5	Sweden	2003	CS	Fjellman-wiklund *et al *[19]
	3.0	7 days							
	2.3 (hip)	12 months	Physical education teachers	359	74.6	Estonia	2002	CC	Pihl *et al*[16]
	3.9 (hip)	12 months	School teachers	359	74.6	Estonia	2002	CC	Pihl *et al*[16]
	14.0 (knees)	12 months	Physical education teachers	359	74.6	Estonia	2002	CC	Pihl *et al*[16]
	7.8 (knees)	12 months	School teachers	359	74.6	Estonia	2002	CC	Pihl *et al*[16]
	8.3 (hips)	12 months	Music teachers	61	58.1	Sweden	1998	CS	Fjellman-Wiklund & Sundelin [18]
	13.9 (knees)	12 months	Music teachers	61	58.1	Sweden	1998	CS	Fjellman-Wiklund & Sundelin [18]
	5.5 (feet)	12 months	Music teachers	61	58.1	Sweden	1998	CS	Fjellman-Wiklund & Sundelin [18]
	13.9 (hips)	12 months	Music teachers	62	98.4	Sweden	1998	CS	Fjellman-Wiklund & Sundelin [18]
	8.3 (knees)	12 months	Music teachers	62	98.4	Sweden	1998	CS	Fjellman-Wiklund & Sundelin [18]
	5.5 (feet)	12 months	Music teachers	62	98.4	Sweden	1998	CS	Fjellman-Wiklund & Sundelin [18]
	33.0	NS	Preschool teachers	22	95.4	US	1995		Grant *et al*[11]

^a ^Recall period (NS = Not Specified)

^b ^Response rate of the study (*Total number of respondents listed as the response rate was not provided)

^c ^Publication year

^d ^Study design (CS = Cross sectional, CC = Case Control)

In more recent studies, MSD prevalence rates have been found to be 68% for music teachers in Perth, Australia [7], and 55% and 51.4% for school teachers in Brazil [3] and Turkey [15], respectively. Music teachers may be at an increased risk for MSD when compared with other school teachers. In comparison, PETs tended to have low risk of MSD, while preschool teachers have been reported to be at an increased risk of MSD [11]. As most of the studies reviewed had examined prevalence in selected musculoskeletal regions, these results will be examined separately.

### Neck and/or shoulder pain

Most studies have measured neck and shoulder pain separately as being 'neck pain' or 'shoulder pain,' although a few have combined them as 'neck and/or shoulder pain'. In a study of secondary school teachers in Hong Kong, the life-long prevalence of neck pain has been reported at 69.3%, within 12 month prevalence of 66.7%, and the prevalence after becoming a teacher being 59.7% [9]. Similar findings have been demonstrated in another Chinese study where secondary school teachers reported a life-long prevalence of neck pain as 68.2%, 64.4% for 12 months, and neck pain prevalence after becoming a teacher of 56.8% [6]. In a more recent Chinese study, school teachers reported a high neck pain prevalence rate of 68.9% for the previous month [14]. Parallels can be drawn to other studies where 59% of Swedish music teachers reported neck pain in the previous 12 months [19]. Furthermore, in a more recent study of Swedish music teachers, 47% reported having experienced neck pain in the previous 12 months [20]. Similar results have been found in another study of Swedish music teachers [18] where 44.4% experienced neck pain. In other studies, 42.5% of Turkish school teachers reported having experienced neck pain [15]. In comparison, PETs reported the lowest neck pain prevalence rate of all, being 9.3% [16].

The highest shoulder pain prevalence (73.4%) for the previous month has been reported by Chinese school teachers [14], while in Turkey, 28.7% of school teachers had experienced MSD symptoms in the shoulder area [15]. Furthermore, the prevalence of shoulder pain varied greatly between 28% and 55% in studies of Swedish music teachers carried out between 1988 and 2009 [16,18-20]. In Estonia, 7.8% of non-PETs and 18.6% of PETs reported pain on their shoulders [16]. In Japan, 25% to 35.4% of preschool teachers had experienced neck and/or shoulder pain in the previous month [21]. Comparable to these findings are the results of a US study in which 33% of preschool teachers reported neck and/or shoulder pain [11].

### Upper limbs/extremities

Several studies have investigated MSD in the upper extremities such as the elbows, wrist, arm or hands. Upper limb pain was reported by 72% of Japanese teachers of physically and intellectually disabled pupils [22], and by 23.7% of Brazilian school teachers [3]. In a Chinese study of secondary school teachers, 35.8% reported life-long upper limb pain whilst 33.3% had experienced upper limb pain in the previous 12 months and 31.8% had experienced upper limb pain since becoming a teacher [9]. Elbow pain has been reported as a symptom, mainly by music teachers. From the Swedish studies carried out among music teachers, the prevalence of elbow pain ranged between 11.1% and 22.2%. Pain in the hand region has also been the most prevalent symptom among Swedish music teachers, ranging from 13 - 22.2% of the teachers surveyed [18-20]. Only 8% of school teachers in Turkey reported elbow pain [15], however, a total of 43.9% of primary and secondary school teachers in Hong Kong reported MSD in the arm during the previous month [14]. In contrast, 9.1% to 17.7% of Japanese preschool teachers reported having experienced arm pain, while 11% of US preschool teachers had experienced hand/wrist pain [11]. Wrist pain was a symptom reported by only 13% of the Turkish school teachers [15].

### Back pain

Many studies examined in the current review had measured back pain in different ways. Most reported back pain in general, while comparatively fewer studies reported low back and upper back pain separately. For example, 63% of Greece PETs [23] and 52.4% of teachers in a special school for the severely handicapped reported back pain [24]. Similar results were found in two studies conducted in Japan where 45.7% of teachers for physically and mentally handicapped children [25] and 76.7% of teachers for physically and intellectually disabled pupils [22] reported higher prevalence rates of back pain. The prevalence of back pain among preschool teachers also varied greatly. In two separate studies of Japanese preschool teachers, 17.7% [26] and 43.3% [5] reported back pain, while a higher prevalence of 61% has been reported among US preschool teachers [11].

In the Philippines and Brazil, 53.3% of secondary school teachers [8], and 41.1% of primary and secondary school teachers [3] have reported back pain, respectively. Parallels can be drawn to other studies where 40.4% of Malay teachers [1] and 40% of Chinese primary school teachers also reported back pain [27] in the 12 months prior to the study. In France, 34.8% of school teachers had experienced back pain in the previous six months [28]. Conversely, only 20.6% of Japanese preschool school teachers had experienced back pain [5].

Lower back pain appears to be more prevalent than upper back pain among teachers. Supporting this hypothesis is a Turkish study which found that 43.8% of school teachers reported low back pain, compared to 36.9% of whom reported upper back pain [15]. Similar results have been demonstrated in a Chinese study where 59.2% teachers reported low back pain compared to 52.5% who reported upper back pain [14]. Furthermore, a number of Swedish studies conducted among music teachers have found similar trends [18-20]. It must be noted, however, that in Estonia, PETs reported a significantly lower prevalence of low back pain (4.7%), when compared to non-PETs (11.8%) [16].

### Lower extremities

A few studies have investigated MSD of the lower extremities such as hips, legs, knees, ankles and/or feet among teachers. MSD in the lower extremities have been reported by 41.1% and 33% of Brazilian school teachers [3] and US preschool teachers [11], respectively. In China, 54.6% of school teachers reported having experienced leg pain during physical activity in the previous month [14]. In a recent Turkish study, lower extremity pain had been experienced by 8.4% of teachers in the hip area, 32% in the knees and 21.8% in the ankles [15]. In another study, 12% of Swedish music teachers reported hip pain, 16% knee pain and 9% foot pain in the previous 12 months [19]. In Estonia, 3.9% of non-PETs reported hip pain in the previous 12 months, whilst 2.3% of PETs reported hip pain over the same time period. In comparison, in the same Estonian study, only 7.8% of non-PETs reported knee pain whilst 14% of PETs reported experiencing knee pain [16]. The prevalence of pain in the lower extremities of teachers seems to be relatively low when compared to the prevalence of pain in the upper extremities and the back.

### Risk factors

#### Individual factors

From the reviewed literature, it appears that the prevalence of MSD is positively associated with female gender. Supporting this hypothesis are the results of a Chinese study, where female teachers experienced neck pain (p < 0.001) and upper limb pain (p < 0.001) more frequently than their male colleagues [9]. Parallels can be drawn to the results of a Turkish study which reported that female teachers are at risk of neck pain (p = 0.001), upper back pain (p = 0.004) and shoulder pain (p = 0.002), when compared to their male counterparts [15]. In addition, it appears that Chinese female teachers have been more likely to report low back (p < 0.01), neck (p < 0.001), shoulder (p < 0.001), upper back (p < 0.001), arm pain (p < 0.001) and leg pain (p < 0.001) during physical activity [14].

Gender appears to be significant when considering the issue of MSD among music teachers. This is supported by the findings of a Swedish study where female music teachers reported a significantly greater number of problems in the neck (p = 0.02), upper back (p = 0.01) and shoulder (p = 0.025), when compared to male music teachers [20]. These results are in agreement with the findings of a study conducted among music teachers in Australia, where 45.9% of females and 33.8% of males reported MSD (p < 0.05) [7]. In Sweden, female music teachers reported significantly more symptoms in the neck (p = 0.02), the shoulders (p = 0.02), the upper back (p = 0.00) and the feet (p = 0.01) [19] than their male colleagues. Contrary to these findings are the results of a Filipino study that did not document any significant gender differences between teachers with and without low back pain (p > 0.05) [8].

Female gender has also been positively associated with the severity of MSD. A study from Turkey, for example, found that female teachers report more severe pain in the wrist (p = 0.044), upper back (p = 0.008) and lower back (p = 0.022) regions [15]. Similar findings have been reported in a study of Chinese teachers, where female teachers experienced a higher pain severity in the shoulder than their male counterparts (p < 0.001) [14].

Conflicting findings have been demonstrated in the relationship between age and MSD. In Brazil, teachers above 40 years of age were more likely to report lower limb pain (Odds Ratio (OR):1.28, 95% CI:1.01-1.38), back pain (OR:1.20, 95% CI:1.07-1.35) and upper limb pain (OR:1.31, 95% CI:1.10-1.56) [3], while a study of Turkish teachers has found that teachers above 40 years were more likely to report MSD (p < 0.001) [15]. In other studies, however, younger teachers have also been found to experience MSD. This has been evidenced in the results of a Chinese study where the age group with the highest prevalence of neck pain was 31-35 years, with a significant difference among different age groups in the prevalence of neck pain (p < 0.001). In the same study, the age groups with the highest prevalence of upper limb pain were 46-50 years and > 50 years, with a significant difference among age groups in the prevalence of upper limb pain (p < 0.001) [9]. In two other Chinese studies, teachers aged 30-39 years had experienced the most low back pain (OR: 1.30, 95% CI: 1.00-1.70) [27], while teachers aged 31-50 years had also reported experiencing upper back pain (p < 0.05) [14].

Length of employment has been significantly associated with neck pain among Chinese secondary school teachers (OR: 1.11, 95% CI: 1.01-1.23) [6], and also with low back pain among Chinese teachers (OR: 1.80, 95% CI: 1.30-2.40) [29]. Among Brazilian teachers, length of employment has been significantly associated with lower limb (OR: 1.12, 95% CI: 1.01-1.19), back (OR: 1.15, 95% CI: 1.07-1.24) and upper limb pain (OR: 1.34, 95% CI: 1.19-1.50) [3]. In Japan, length of employment has been associated with pain in the neck/shoulders (OR: 1.37, 95% CI: 1.15-1.64) and arms (OR: 1.65, 95% CI: 1.30-2.08) in nursery school teachers [21].

Long working hours have also been significantly associated with MSD. A strong correlation has been reported between low back pain and Greek PETs who spent more than 35 hours per week teaching physical education (p < 0.05) [23]. In Brazil, working more than 40 hours a week has been associated with pain in the upper and lower limbs (p < 0.05) [3]. Having more than 30 students in a class has been positively associated with upper limb pain among Brazilian school teachers (p < 0.05) [3]. Intensive physical activity in leisure time has been correlated with increased knee pain (p < 0.01) among Estonian PETs [16].

#### Physical factors

In the US, reduced playing time, having smaller hands and lower strength levels has been associated with MSD among music teachers [17]. Kneeling, stooping, squatting and bending have been significantly associated with MSD among US [11] and Japanese preschool teachers [29]. Intense physical exertion (Prevalence ratio (PR):1.29, 95% CI: 1.20-1.38) and inappropriate furniture (PR: 1.11, 95% CI: 1.03-1.19) have also been positively associated with back pain among Brazilian teachers [3]. High participation in lifting, especially when supporting students on gymnastics apparatus [30], and high participation in sports among Swedish PETs have been shown to be highly correlated to knee pain [31].

#### Psychosocial factors

Various studies have reported that poor psychosocial factors were potential risk factors for MSD. In a Chinese study of secondary school teachers, low colleague support (OR: 2.00, 95% CI: 1.16-3.47) and high workload (OR: 2.17, 95% CI: 1.58-2.97) have been significantly associated with neck pain [9]. Other studies have also demonstrated a significant association of psychosocial factors and MSD [5,6,21]. Furthermore, psychosocial factors such as mental health among Malay school teachers (OR: 1.11, 95% CI: 1.06-1.15) [1] and anxiety among Chinese teachers (OR: 1.49, 95% CI: 1.07-2.07) [9] have been associated with higher MSD prevalence rates.

## Discussion

### Assessment of MSD

Overall, this review suggests that while MSD is most likely an under researched topic among teachers, teaching itself represents a high risk occupation for MSD. The findings of this literature review have been drawn from 33 papers, each of which had measured different musculoskeletal regions using different methods. As most studies had used self-developed questionnaires [3,7,15,21-23,26,28], or the Standardized Nordic Questionnaire [1,18,27], it appears that these are commonly accepted methods for measuring the prevalence of MSD. Other methods used included pilot tested surveys and questionnaires such as the Northwick Neck Pain Questionnaire [6,9], Health Questionnaires [25], Job Content questionnaires [5] and the Subjective Health Complaints Questionnaire [14]. While questionnaires are an inexpensive and convenient mode of data collection, they can introduce recall bias and make follow up difficult, especially when anonymous reporting is utilised. More accurate results might be obtained by physical examination and assessment, although these methods are expensive and time consuming, and therefore, ultimately uncommonly seen in the literature.

### Response rate

The response rate among most of the studies examined in this review was acceptable, although one investigation reported a response rate of only 3.5% [17]. For this study, the participants had been recruited during a conference using a poster placed near the main conference rooms. Some conference attendees might not have seen the poster while others might have been too busy with the conference proceedings to participate, consequently leading to the low response rate.

### Prevalence

The most prevalent body regions on which teachers reported MSD have been the back [1,3,5,8,14-16,18-20,22-28], neck [6,9,14,15,18-20] and upper limbs [3,9,14,15,18-20,22]. It is important to note that while a number of studies have been carried out to specifically investigate back and neck pain, few studies have specifically looked at whole body MSD, and no studies have been carried out to specifically investigate lower extremity MSD. It is important, however, to note that the reported prevalence of back pain varied greatly across the literature, ranging from 4.7% to 76.7%.

Teachers of physically disabled pupils have reported the highest back pain prevalence [22] and this might be attributed to the lifting of the disabled pupils. On the other hand, PETs had reported the lowest back pain prevalence, and this may be because PETs are physically active and may also be involved in leisure-time physical activity [16]. However, the absence of personal training in order to maintain physical fitness among PETs could contribute to low back pain [23]. PETs have been reported to have a higher prevalence of knee disorders than non-PETs and were more likely to change work due to knee dysfunction [31].

### Individual factors

MSD among teachers has been positively associated with female gender in a number of studies. It has been suggested that women might be more likely to report pain than men because women have lower physical strength, pressure from family and career prospects; or simply the fact that men and women have different traditions and thresholds for when and how they report pain [14]. While MSD has been positively associated with length of employment, research findings are somewhat inconsistent with some studies reporting longer length of employment as being positively associated with MSD, while others have reported that new teachers are more likely to report MSD. It has also been reported that the longer the exposure time to occupational risk factors, the higher the possibility of incurring job-related disorders [9]. This association can be interpreted as the effect of aging or a cumulative effect of workload on the musculoskeletal system of workers [21]. Where teachers with lesser teaching experience had reported MSD, it has been suggested that this may occur because new teachers might not be adapting well to the new working environment, and that physical and psychological stress might be affecting the wellbeing of their musculoskeletal conditions [9]. Further studies will be needed to investigate such a hypothesis.

### Physical factors

The use of a 'head down' posture has been significantly associated with neck pain (OR: 2.17, 95% CI: 1.38-2.74) and this may impact on teachers who spend considerable time correcting students' work [9] and preparing for lessons. Neck pain among teachers has been positively correlated with computer processing posture [9]. It has been hypothesised that working with a 'poking chin' posture during computer processing might induce considerable load on the posterior, leading to increased loading on non-contractile structures and posterior cervical structures, thereby resulting in neck pain [9].

### Psychosocial factors

Psychosocial factors have been positively associated with MSD among school teachers, and the current review suggests that psychosocial factors such as high workload/demands, high perceived stress level, low social support, low job control, low job satisfaction and monotonous work are most likely associated with MSD among school teachers [1,5,6,9,21]. This may occur because teachers often work in stressful conditions with large classes, a lack of educational resources, and limited reward for their work [3].

### Limitations

A number of limitations were identified in this review. Recall bias and self-reporting can be considered as limitations for a number of studies, given that many used anonymous survey for data collection [3,7-9,15,19,20,25,27,28,32,33]. Cause and effect inferences cannot be ascertained, however, given that a number of studies used a cross-sectional study design [3,7-9,19,27]. Sample sizes and response rates were suboptimal in some studies [14,17,18]. Additionally, it must be noted that the review considered articles that were written in English only.

## Conclusion

This literature review clearly suggests that teachers are at risk for developing MSD. The prevalence among them is not uniform; however, music teachers have been known to retire before their retirement age due to MSD. Primary and secondary school teachers appear to be more prone to neck, shoulder and back pain. Further studies, preferably longitudinal, are required to more thoroughly investigate MSD among teachers, with a greater emphasis on ergonomic factors. This would represent a major step forward in prevention of MSD among teachers, especially if easy to implement control measures could be recommended.

## Competing interests

The authors declare that they have no competing interests.

## Authors' contributions

PNE conceived and designed the study and carried out data collection and analysis. PNE and DRS read and approved the final manuscript.

## Pre-publication history

The pre-publication history for this paper can be accessed here:

http://www.biomedcentral.com/1471-2474/12/260/prepub
